# The effects of involvement in training and volunteering with families of people with dementia on the knowledge and attitudes of volunteers towards dementia

**DOI:** 10.1186/s12889-022-12687-y

**Published:** 2022-02-08

**Authors:** Daphne Sze Ki Cheung, Lily Yuen Wah Ho, Robin Ka Ho Kwok, Daniel Lok Lam Lai, Claudia Kam Yuk Lai

**Affiliations:** 1 School of Nursing, The Hong Kong Polytechnic University, Hung Hom, Kowloon, Hong Kong; 2 The Hong Kong Hospital Authority, Hospital Authority Building, 147B Argyle Street, Kowloon, Hong Kong

**Keywords:** Volunteer, Dementia knowledge, Dementia attitudes, Motivation to volunteer, Training, Service, Public health

## Abstract

**Background:**

Volunteers have been a valuable resource in supporting people with dementia and their caregivers in the community. However, factors such as misconceptions, negative attitudes towards dementia, and a lack of motivation might impact the quality of volunteer care. The present paper aims to examine the effect of training and service provision on the knowledge and attitudes of volunteers towards dementia and the association between knowledge and attitudes with the levels of motivation to volunteer.

**Methods:**

The present study is part of an effectiveness-implementation cluster randomized clinical hybrid trial using a music-with-movement intervention to promote the well-being of people with dementia and their informal caregivers. Volunteers were recruited to receive training to support the delivery of the intervention. Training and enrichment workshops were offered to volunteers during the one-year project. Before and after their training and service, the recruited volunteers were asked to complete the following assessments: Dementia Attitudes Scale, and the Alzheimer’s Disease Knowledge Scale. The levels of motivation to volunteer was measured with Volunteer Functions Inventory at baseline. Wilcoxon signed-rank test and multiple regression test were applied for statistical analyses.

**Results:**

A total of 107 volunteers were recruited, and 81 of them completed a mean period of 47.32 weeks of training and service. Significant improvements in their total score on the Alzheimer’s Disease Knowledge Scale (*p* = .009) and Dementia Attitudes Scale (*p* < .001) were found. Dementia attitude (β = .57, *p* < .001) and knowledge (β = -.18, *p* = .038) were found to have the most significant association with the levels of motivation to be a volunteer at baseline.

**Conclusions:**

The present study illustrated the importance of quality volunteer training and voluntary service in improving the dementia knowledge and attitudes of volunteers. It also shed light on the association between knowledge and attitudes with the levels of motivation to volunteer. Accordingly, future research and public health policymakers should address more efforts to amplify the advantage of volunteers as a vital asset in dementia care.

**Trial registration:**

NCT03575026 (ClinicalTrials.gov), First registration on 02/07/2018.

## Background

Volunteering, which is a helping behavior, refers to the provision of free services to benefit other people or organizations [1]. Volunteers are important additions to dementia care. Currently, there are about 50 million people in the world have dementia, and a proliferating number of nearly 10 million new cases can be expected each year, which will squarely challenge the planning framework for community healthcare practice and implementation [2]. Policymakers will unavoidably have to develop strategies and improve capacity to address these current and future impacts of dementia as an increasing threat to global public health. One of the approaches is to increase the number of volunteers and their involvement. They are valued as extra resources to providing many aspects of care services and serve as adjunct manpower to supplement the work of regular healthcare providers [3].

Volunteers have been mobilized to serve in various assistive roles in dementia care with positive outcomes. For instance, shorter lengths of stay in acute hospitals were found among patients with dementia who received person-centered care from volunteers that focused on nutrition and hydration support, help with hearing and visual aids, activities, and orientation [4]. In the community and long-term care settings, people with dementia were also found to have benefited from volunteer-administered non-pharmacological interventions [5, 6]. The benefits of including volunteers in the care team are not limited to people with dementia but also extended to their caregivers. Family caregivers and hospital staff reported reduced stress and burden when volunteers were integrated into the care team providing person-centered care, and stated the contribution of volunteers to their quality of care [7]. Apparently, volunteers can improve the outcomes of both people with dementia and their caregivers, especially in systems with scarce resources.

The main reason to engage in volunteering in the community tends to be altruistic [8], and yet volunteers benefitted from their role in a reciprocal manner. Previous studies have found that volunteering is associated with improved functioning [9], better self-reported health [10], greater feelings of purpose in life [11], higher levels of life satisfaction [12], and decreased risk of mortality [13]. Evidence has also shown that volunteers acquired relevant knowledge after providing voluntary services [14]. However, there is relatively less discussion in the literature about the benefits of volunteering in the area of dementia care services, with quantitative studies exceptionally scarce [15]. In qualitative studies, volunteers for caregivers of people with dementia verbally expressed their enjoyment and satisfaction from their role, increased awareness of the challenges faced by the families, and enhancement in self-understanding [16, 17].

Little is known about or quantifying their gains in knowledge and changes in attitudes towards dementia after training and providing services to families of people with dementia. Nevertheless, preliminary evidence suggested healthcare professional trainees’ perceived improvement in dementia knowledge and more confidence in engaging with people with dementia, after attending the Dementia Friends educational program [18]. The content of the program included information about dementia and effective communication techniques, and advocated the five key messages (dementia is not a natural part of ageing; dementia is not just about memory loss; dementia is caused by diseases of the brain; it is possible to live well with dementia; and there is more to the person than the dementia) [19]. In contrast, there are some critiques that the Dementia Friends training may fall short of achieving its goals of improving attitude and among general public, although it is successful in terms of reach and impact [20]. There is a pressing need for further evaluating the effectiveness of volunteer programs on dementia knowledge and attitude.

Improving the knowledge and attitudes of the volunteers towards dementia can contribute to several demonstrable benefits to the general population. First, increasing dementia knowledge and correcting misconceptions may help overcome the stigma and create a dementia-friendly environment [21, 22], which is one of the important strategic directions initiated by the World Health Organization [23]. Second, while many perceive dementia as inevitable and non-preventable [24], trained volunteers can be at the forefront of the preventative measures to bring their knowledge and experiences back to their local communities and improve public awareness about the range of management associated with dementia. Third, most middle-aged and older individuals were unaware of the dementia risk [25]. Educating the middle-aged or older volunteers may help prevent delays in the help-seeking process when they had dementia symptoms [26]. Therefore, it is important to identify an effective way to enhance knowledge and attitudes towards dementia, particularly among volunteers.

Traditional didactic teaching methods that offer “one-off” training for volunteers before sending them off to serve might not be effective at improving their knowledge and attitudes, such as the Dementia Friends program [20]. This is because such an approach does not offer opportunities for “real learning” to take place, as learning is a continuous process of reflective observation, abstract conceptualization, active experimentation, and the acquisition of concrete experiences [27]. Furthermore, issues such as a decline in motivation and dropping out from volunteering are also encountered in “one-off” training. A recent study found that people could feel more negatively about people with dementia after exposure to alarming symptoms [22]. Moreover, volunteers have often been found to be struggling to manage the discrepancy between their expectations of volunteering and their “actual” experience of it [28]. Therefore, the research team designed a training program that incorporated elements of continuous learning, where volunteers were allowed to reinforce their lectured knowledge and skills by having contact and interacting with people with dementia and their family members. Their training needs were to be fulfilled by actively engaging them in designing the training content. Better dementia knowledge and attitudes may motivate the volunteers to serve and yet to be confirmed. Hence, the research problem that we investigated was whether a specially designed training program for volunteers such as the one we used in our study would effectively enhance their knowledge and attitudes towards dementia. An attempt is also made to investigate the association between dementia knowledge and attitudes with the levels of motivation to volunteer.

### Objectives and hypotheses

The objectives and the related hypothesis of this project were:

- Objective 1: To examine the effect of training and service provision on the knowledge and attitudes of volunteers towards dementia.
- Hypothesis 1: The volunteers would have significant improvement in the knowledge and attitudes towards dementia after training and service provision.
- Objective 2: To identify the association of dementia knowledge and attitudes at baseline with the levels of motivation to volunteer at baseline.
- Hypothesis 2: Dementia knowledge and attitudes would have positive and significant association with the levels of motivation to volunteer.

## Methods

### Design

This study was a part of an effectiveness-implementation cluster randomized clinical hybrid trial (ClinicalTrials.gov Identifier: NCT03575026) to evaluate the effectiveness and implementation of a music-with-movement intervention for enhancing the well-being of both people with dementia and their informal caregivers. The data collected from the volunteers were analyzed to address the research objectives. This study is reported according to the Transparent Reporting of Evaluations with Nonrandomized Designs (TREND) guideline [29].

### Participants and setting

This study was conducted in Hong Kong SAR, China. Participants were recruited through convenience sampling by the seven partner organizations and through an institute of the University consisting of 4,000 senior members. The seven partner organizations provide elderly services (*n* = 4), women’s services (*n* = 1), mental health services (*n* = 1), or general social services (*n* = 1).

The criteria for the inclusion of participants were (1) aged 18 and above; (2) physically stable to pay home visits; (3) able to speak Cantonese (a major dialect in Hong Kong) and write Chinese; and (4) without cognitive impairment or psychiatric illness. Those who could not commit to volunteering for families of people with dementia without pay for at least six months were excluded.

### Training and enrichment workshops

Five 2-h mandatory workshops and ten optional enrichment workshops were arranged for all the recruited participants and were conducted in the University. Each of the five mandatory workshops are delivered in different small groups that had around 15 – 20 participants to facilitate more direct instructional support in enhancing their learning performance, and they were provided with the intervention manual and teaching handouts. The first mandatory workshop delivered by a gerontological nursing scientist was about dementia, interaction with people with cognitive impairment, and the practical skills for leading the music-with-movement intervention. Case scenarios were shared and discussed to facilitate reflection and problem solving among the participants. The second to fifth mandatory workshops were delivered by a registered music therapist. Of these four workshops, the first hour was conducted with around 3 – 5 staff working in the partner organization. The volunteer participants had a roleplaying attempt that tried to play a role similar to that of the clients with dementia, in that they received music-with-movement interventions led by the center staff who would adopt the intervention in the working site. The second hour focused on the theoretical and practical knowledge related to music-with-movement interventions, and the skills required to lead the activities. The details of the activities designed for people with dementia can be found in the published E-book: https://lihi.cc/XItEY. Throughout the sessions, the skills involved in communicating and empathizing with people with dementia were regularly reinforced. This kind of information may help the participants to understand the symptoms of cognitive impairment and may lead them to adjust their preconceptions.

At the end of the five sessions, volunteers were assessed by the music therapist and research team through return demonstration to ensure they were competent. The assessment components consisted of skills in delivering music-with-intervention to people with cognitive impairment (for example, choosing music genre, matching movement to the music, and safety measures); and communication skills (such as speaking speed, clarity of instruction, friendliness, and techniques to lead activities). Those who attended at least 80% of the training sessions and who passed the assessments were recognized as qualified.

Apart from the mandatory training workshops, 10 optional enrichment workshops (2 – 3 h each) were held for all volunteers during the one-year period of the project. No additional assessment after these ten workshops was deemed necessary, as this was the strategy for engaging the volunteers to support the project continuously and further advancing their knowledge. The topics of these enrichment workshops were suggested by the volunteers and partner organizations. The details of the content of the workshops are listed in Table 1.

**Table 1:** Content of the mandatory training and enrichment workshops

Topic (*n* = number of attendees)	Speaker background	Key content
Mandatory training workshop		
Session 1: Understanding cognitive impairment (*n* = 107)	Gerontological nursing scientist	•Introduction of the project •Symptoms of cognitive impairment •Normal ageing and cognitive impairment •Communication skills and activity leading skills •Initial management for behavioural and psychological symptoms of dementia •Building rapport with the clients •Anticipated difficulties
Session 2–5: Practical skills for leading music-with-movement intervention (*n* = 107)	Registered music therapist	1^st^ hour: •Experience the music-with-movement intervention as the clients to be served 2^nd^ hour: •Choice of music genres •Safety measures •Design of movement •Props to be used
Enrichment workshops		
1. Neuropsychiatric symptoms of dementia and the sharing of volunteer experiences (*n* = 50)	Gerontological nursing scientist	•Common neuropsychiatric symptoms of dementia •Management strategies •Difficulties anticipated during the home visits and solutions
2. Interior design principles of a home for people with dementia (*n* = 16)	Gerontological nursing scientist	•Home safety •Cognitive symptoms and environment •Principles of interior design for people with cognitive impairment
3. A mindfulness-based intervention for caregivers (*n* = 25)	Mindfulness practitioner	•Stress of caregivers •Mindfulness thinking and our brain •Mindfulness practices
4. Stretching exercises (*n* = 14)	Physiotherapist	•Stress and exercises •Practices of stretching exercises
5. Reminiscence for older people with dementia (*n* = 15)	Social worker	•What is reminiscence •Reminiscence activities for older people
6. Emotional support to caregivers (*n* = 5)	Social worker	•Caregiver burden •Community services available in Hong Kong •Basic counselling techniques
7. Zen drawing for stress reduction (*n* = 19)	Activity worker	•Benefits of Zen drawing •Zen drawing practices
8. Site walk: Technology for older people with dementia (*n* = 5)	Social worker	•Introduction to the services and facilities of a new center
9. Breathing exercise (*n* = 23)	Activity worker	•Stress and breathing exercises •Practice breathing exercises
10. Mental health (*n* = 10)	Social worker	•Common caregivers mental health problems •Strategies to promote mental health

Instant messaging had been proved to empower the team and facilitate knowledge sharing in a workplace that heightened team performance [30]. We anticipated volunteers might share a commonality of interest. Therefore, instant messaging groups (using WhatsApp) were formed to facilitate support by the project team (including the music therapist) and center staff to volunteers. Each partner organization was a unit of the chat group.

### Voluntary services to families with people with dementia

After the volunteers had received the five sessions of training and satisfying the assessment criteria (i.e. the skills in delivering music-with-intervention to people with cognitive impairment; and communication skills), they were arranged to meet the families in the center. There were no restrictions on how frequently they should contact the families, but it was recommended that they visit the home of the families at least once a month during the 24 weeks of the intervention program. Two volunteers were paired up and assigned to serve one or two families. During the home visits, the two volunteers were expected to deliver the 30 – 45 min music-with-movement intervention to the clients with cognitive impairment, and to communicate with the family member to solve problems related to the intervention. Each session of the music-with-movement intervention consisted of four to five designed music activities, such as tapping the feet with music and singing [31]. During these visits, the volunteer also observed the people with dementia and the family’s interaction, and helped address questions from the families, if any, on the areas they had been trained to address. The matching was carried out by the staff of the center, who was familiar with the background of both the clients and the volunteers. The principles in conducting the matching were the geographical proximity of the client and the volunteers’ homes and the preferences (e.g. female volunteer) of the client and volunteer.

The volunteers were also required to assist the staff members in delivering four center-based, group-based, music-with-movement interventions in the first 12 weeks, for three purposes: (1) to assist the staff in the activities; (2) to observe the professional interaction between the staff members and people with cognitive impairment and their family members; and (3) to practice the skills under the supervision of the trained staff and project team.

During this period of the project, apart from the communication via the instant messaging group, the volunteers were required to record their experiences within one week after each visit in a standardized written form, which include the documentation of the family members in delivering the intervention and the volunteers’ personal feeling in their role of supporting. The project team reviewed the volunteers’ electronic records and swiftly answered any queries that they made regarding the visits. The volunteers also shared their experiences during the ten enrichment workshops, where the professional speakers also answered questions. The quality of the volunteers’ services was ensured in the abovementioned ways.

### Outcome measures

Participants were asked to fill in a questionnaire at the training venue, without discussing it with their peers. Although the survey was designed to allow for self-administration, a research assistant was trained to assist those who might need assistance (e.g., those who could not read clearly because of presbyopia). Information on demographic characteristics (including age, gender, level of education, employment status, and dementia caregiving experience) was collected at baseline after giving the consent on the first training day. After the completion of training and services provision, participants were asked to fill in the post-test survey.

To address Objective 1, knowledge and attitudes were assessed using the Alzheimer’s Disease Knowledge Scale (Chinese version) and the Dementia Attitudes Scale (Chinese version) respectively at baseline (i.e., before joining any workshops) and within two weeks after the completion of the training and voluntary service provision (expected to be after the 41^st^ week after serving two cohorts of the family). The Alzheimer’s Disease Knowledge Scale [32] consists of 30 true/false items covering the life impact, risk factors, symptoms, treatment and management, assessment, caregiving, and course of the disease. Higher scores indicate more correctly answered items. The test takes approximately 5 – 10 min to complete. The internal consistency (Cronbach α = 0.71) and test–retest reliability (*r* = 0.81) of the scale were acceptable and good, respectively [32]. The Dementia Attitudes Scale [33, 34] consists of 20 items rated on a 7-point Likert scale (ranging from 1 to 7) that reflect the affective, behavioral, and cognitive components of attitudes towards individuals with Alzheimer’s and related forms of dementia. Two factors were identified, namely, dementia knowledge and social comfort. The internal consistency was good (Cronbach α = 0.83 – 0.85) [33].

To understand the levels of motivation to be a volunteer and explore its relationship with dementia knowledge and attitudes (Objective 2), the participants were asked to fill in the Volunteer Functions Inventory (Chinese version, [35]) at baseline. The inventory contains 30 items and six subscales (with 5 items each) rated on a 7-point Likert scale (ranging from 1 – 7), with higher scores indicating the greater importance of that motive. The subscales measure six major functions potentially served by volunteerism: reducing one’s negative feelings (protective), expressing a generous concern towards others (values), gaining career-related experience (career), strengthening one’s social relationships (social), learning through hands-on experience (understanding), and promoting better feelings towards oneself (enhancement). The internal consistency of each subscale was found to be good to excellent (Cronbach’s α = 0.70 – 0.91), with the test–retest reliability over an 8-month interval ranging from *r* = 0.56 to 0.73 [35].

### Sample size

As this was part of an implementation study, a formal estimation of sample size was not performed for addressing the abovementioned research objectives. However, the amount of funding allowed the team to serve 75 families, and therefore we anticipated to recruit at least 75 volunteers to serve the recruited families.

### Ethical considerations

Approval for the study was obtained from the University (HSEARS20180319002). Written consent was obtained from all participants. To compensate them for their transportation costs, the participants received the equivalent of about US$7.7 in cash per trip at the end of the program. The conduct of the study was consistent with the requirements of the National Statement on Ethical Conduct in Human Research.

### Statistical analysis

Data were analyzed using SPSS software version 25.0. A Wilcoxon signed-rank test was used to examine the change in dementia knowledge and attitudes towards dementia after training and voluntary service provision. Hierarchical multiple regression was used to assess whether dementia knowledge and attitudes could predict an individual’s levels of motivation to volunteer, after controlling for possible confounders (i.e., age, gender, level of education, employment status, and dementia caregiving experience). The level of significance was set at *p* < 0.05 (two-tailed) in all statistical analyses.

## Results

**Fig. 1:**
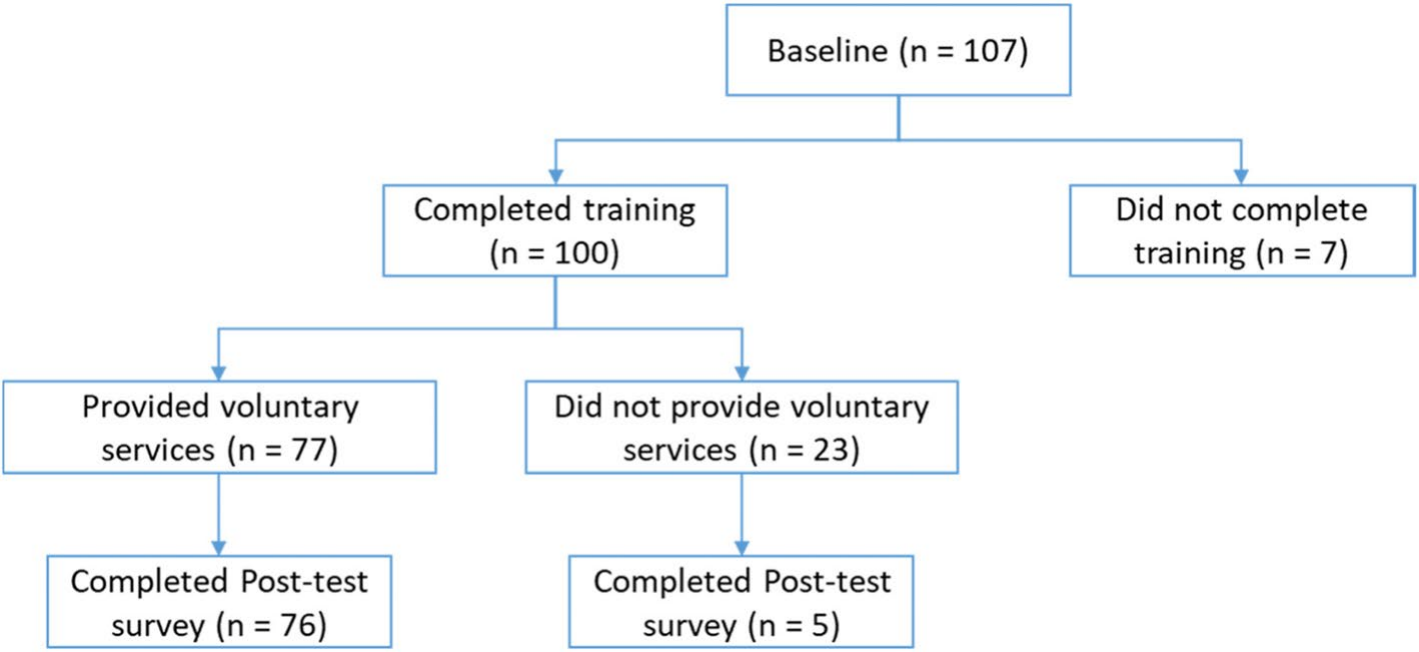
Participant flow chart

A total of 107 volunteers were recruited in this study in the period of March – August 2018, and 81 of them completed the program by completing the post-test survey not later than October 2019 (see Table 2 and Fig. 1 for details). The majority were female (90.65%) and unemployed or retired (73.83%). Around half of the sample were older than 60 (48.60%), with a mean age of 58.07. One hundred participants (93.46%) attended over 80% of the training workshops and were eligible to provide voluntary services. Around 70% of the volunteers did not have any experience in caring for people with dementia before the training. The mean period between the baseline assessment and the follow-up was 47.32 weeks (SD = 6.40).

**Table 2:** Characteristics of the participants

Participants (*n* = 107)	Mean (S.D.)
Age	58.07 (9.21)
Years of education	12.42 (4.39)
Number of visits provided	5.04 (4.94)
Volunteer Functions Inventory (range)	
Protective (5–35)	21.76 (6.52)
Values (5–35)	27.46 (4.92)
Career (5–35)	22.67 (7.06)
Social (5–35)	24.60 (6.18)
Understanding (5–35)	26.69 (5.18)
Enhancement (5–35)	23.97 (6.54)
Total score (30–210)	147.56 (32.81)
	Count (%)
Sex	
Male	10 (9.35)
Female	97 (90.65)
Age > = 60	
No	55 (51.40)
Yes	52 (48.60)
Employment status	
Unemployed/retired	79 (73.83)
Part-time/temporary	2 (1.87)
Full-time	26 (24.30)
Attended over 80% of the training workshops	
No	7 (6.54)
Yes	100 (93.46)
Experience in caring for people with dementia	
No	74 (69.16)
Yes	33 (30.84)
Training workshop attendance	91.21 (14.32)
Optional enrichment workshop attendance	16.64 (17.85)

### Effects on dementia knowledge

The results of the Wilcoxon signed-rank test indicated that there was a significant improvement after the provision of training and voluntary services in the total score of the Alzheimer’s Disease Knowledge Scale (*p* = 0.009), and of the Risk factor (*p* = 0.001) and Caregiving (*p* = 0.032) subscales. The changes in the other subscales did not reach the level of statistical significance (see Table 3 for details).

**Table 3:** Dementia attitudes and knowledge pre- and post-test

	Baseline (*n* = 107)	Post-test (*n* = 81)	*P*-value^a^
	Mean (S.D.)		
Alzheimer’s Disease Knowledge Scale (range)			
Life impact sub-score (0–3)	2.26 (0.79)	2.47 (0.74)	.102
Risk factor sub-score (0–6)	2.80 (1.31)	3.47 (1.28)	**.001**
Symptom sub-score (0–4)	2.71 (1.18)	3.01 (1.14)	.092
Treatment and management sub-score (0–4)	2.93 (1.02)	3.14 (0.95)	.141
Assessment and diagnosis (0–4)	2.52 (0.88)	2.47 (0.98)	.591
Caregiving sub-score (0–5)	2.44 (1.10)	2.84 (1.12)	**.032**
Course sub-score (0–4)	2.26 (0.99)	2.26 (0.96)	.350
Total score (0–30)	17.93 (4.44)	19.44 (4.56)	**.009**
Dementia Attitudes Scale (range)			
Comfort sub-score (10–70)	40.06 (5.29)	41.69 (6.65)	**.003**
Knowledge sub-score (10–70)	53.90 (7.17)	54.58 (6.63)	**.034**
Total score (20–140)	94.05 (10.28)	96.27 (10.70)	**<.001**

^a^Wilcoxon signed-rank test

### Effects on attitudes towards dementia

The results of the Wilcoxon signed-rank test showed that after the provision of training and voluntary services, there was a significant improvement in the total score of the Dementia Attitudes Scale (*p* < 0.001), and of the Social comfort (*p* = 0.003) and Knowledge (*p* = 0.034) subscales (see Table 3 for details).

### Changes of dementia knowledge and attitudes towards dementia between those provided and did not provide services

The Mann–Whitney U-test showed significant differences in the changes score of Alzheimer’s Disease Knowledge Scale (*p* = 0.011) and Dementia Attitudes Scale (*p* = 0.006) between those provided and did not offer volunteering services after training (See Table 4 for details). A post-hoc multiple regression was conducted to investigate if the magnitude of knowledge and attitude change was affected by the number of services delivered, attendance in training workshops and optional enrichment workshops, among those who were eligible to provide services (i.e. attended 80% of the training workshop and passed the assessment). Insignificant regression equations were found for both dependent variables: (1) knowledge change magnitude: F (3,73) = 2.028, *p* = 0.117, adjusted R^2^ = 0.039; (2) attitude change magnitude: F (3, 73) = 0.420, *p* = 0.739, adjusted R^2^ of -0.023.

**Table 4:** Mann–Whitney U-test comparing the change of knowledge and attitudes towards dementia between those provided and did not provided voluntary services

	Did not provide services (*n* = 30)	Provided services (*n* = 77)	Mann–Whitney U	Z	*p*-value^a^
	Median [IQR]				
Alzheimer’s Disease Knowledge Scale					
Life impact sub-score	0 [0 – 0]	0 [0 – 1]	927.00	-1.82	.069
Risk factor sub-score	0 [0 – 0]	0 [0 – 1]	890.50	-1.99	**.046**
Symptom sub-score	0 [0 – 0]	0 [0 – 1]	945.00	-1.72	.085
Treatment and management sub-score	0 [0 – 0]	0 [0 – 1]	995.50	-1.25	.212
Assessment and diagnosis	0 [0 – 0]	0 [0 – 0]	1138.00	-0.14	.891
Caregiving sub-score	0 [0 – 0]	0 [0 – 1]	953.00	-1.57	.116
Course sub-score	0 [0 – 0]	0 [-1.0 – 0.5]	1095.50	-1.57	.645
Total score	0 [0 – 0]	0 [0 – 3]	806.00	-2.56	**.011**
Dementia Attitudes Scale					
Comfort sub-score	0 [0 – 0]	0 [-1.5—6]	1101.00	-0.39	.697
Knowledge sub-score	0 [0 – 0]	0 [-2.5—5]	838.00	-2.24	**.025**
Total score	0 [0 – 0]	0 [0 – 10]	769.50	-2.73	**.006**

### Association between dementia knowledge and attitudes and the levels of motivation to volunteer

The results of the hierarchical multiple regression showed that there was no violation of the assumptions of normality, linearity, multicollinearity, and homoscedasticity. Age, gender, employment status, dementia caregiving experience, and years of education were entered in Step 1, and explained 2.8% of the variance in the levels of motivation to volunteer. After dementia knowledge and attitudes were entered in Step 2, the total variance explained by the model as a whole was 27.8%, F (8, 98) = 6.10, *p* < 0.001. In the final model, only dementia knowledge (total score of the Alzheimer’s Disease Knowledge Scale) and attitudes (total score of the Dementia Attitudes Scale) were statistically significant, with a higher standardized beta value (β = 0.57, *p* < 0.001) for dementia attitudes than for dementia knowledge (β = -0.18, *p* = 0.038) (See Table 5 for details).

**Table 5:** Hierarchical multivariate regression analysis of motives for volunteering at baseline

Constant	**Model 1 β [95% CI]**	***p*-value**	**Model 2 β [95% CI]**	***p*-value**
		< .001		.918
Age	-.01 [-.78, .72]	.936	.03 [-.54, .72]	.771
Female	.05 [-18.46, 28.70]	.667	.01 [-19.14, 20.46]	.948
Years of education	-.05 [-1.98, 1.17]	.612	.01 [-1.25, 1.42]	.896
Employment				
Retired/unemployed (reference)	1		1	
Part-time	.03 [-41.13, 54.52]	.782	-.08 [-60.37, 21.24]	.344
Full-time	-.08 [-21.61, 10.17]	.477	-.13 [-22.89, 3.85]	.161
With dementia caregiving experience	.12 [-5.92, 22.79]	.246	-.01 [-13.38, 12.17]	.925
Dementia knowledge (Baseline)			-.18 [-2.57, -.07]	**.038**
Dementia attitudes (Baseline)			.57 [1.26, 2.38]	** < .001**
**Adjusted *R***^**2**^	**-.030**		**.278**	

## Discussion

To the best of our knowledge, this is one of the first studies to evaluate the impact of volunteer training and voluntary services on the dementia knowledge and attitudes of volunteers. The results showed that the volunteers’ knowledge and attitudes towards dementia had improved after the provision of training and services. These improvements can be attributed to the support we offered through workshops and effective communication with the volunteers that sustain their engagement with the families and strengthen their volunteering experience where actual learning with the targeted clients can take place. Besides, the Mann–Whitney U test results showed that those who provided services had greater improvement in their knowledge and attitudes than those who did not provide volunteering services after training. Supported by the Mann–Whitney U test results, we postulated that the volunteers might actively experiment with and consolidate what they had learned during their visits, and yet to be confirmed in the later research. This experiential learning process might be what helps to reinforce knowledge of dementia (e.g., about the types and progression of the disease and the support needs of people with dementia and their families). This was evident in the significant changes that were observed in the caregiving subscore of the Alzheimer’s Disease Knowledge Scale. In this study, almost 30% of the trained volunteers did not deliver the services mainly for two reasons. First, some families declined the visits as they thought that their flat size was too small for visitors. Second, the center-based activities to be led by the staff did not fit their schedule. Therefore, these practical concerns need to be addressed in the future in order not to disappoint them and a waste of resources.

In addition, we adopted various teaching approaches in our curriculum, such as the use of a written manual; experiential learning workshops and real practices (i.e., services supported by the project team); and interaction with professional staff for modeling the skills. Our approach is similar to that taken in another study on evaluating a curriculum to prepare volunteers to support older persons living with serious illnesses, which included a learning manual, case studies, role-playing, and active engagement with the families [36]. The authors of that study also found that the volunteers were satisfied with this model of training and that they experienced improvements in their self-efficacy. Therefore, we suggest that multiple approaches to volunteer training should be adopted.

Second, the attitude of the volunteers towards dementia improved in this study. The results are consistent with those of another study, which showed that participation in community activities significantly improved attitudes towards dementia [37]. Those without any experience with people with dementia always underestimate the potential of such people due to the negative impressions of them that they receive from society [16]. Through interactions with people with dementia during the assigned activities, the volunteers might have been able to identify and appreciate the ability of people with dementia [38, 39]. They may have witnessed the hardships faced by people with dementia and learned to be more accepting and appreciative of them [39]. The result was a better understanding that led to empathy and sympathy [39].

Regular enrichment workshops were also provided to retaining the volunteers, with topics suggested by the volunteers or participating organizations. An integrative review showed that continuous, ongoing educational workshops and training sessions are a useful strategy to reduce the possible stress experienced by the volunteers, particularly when serving vulnerable groups (such as elderly people and those living in a hospice) [40]. Continuous support offered to the volunteers might have helped them to address the expectations and negative feelings that might arise from volunteering, which are believed to be crucial factors that influence volunteers to drop out [28]. Furthermore, our study showed that the levels of motivation in volunteering was associated with volunteers’ knowledge and attitudes. Knowledge acquired in training and regular meetings enhanced their competence (e.g., communication skills) and confidence that may drive them to continue to volunteer, because of the increased intrinsic motivation [39, 41, 42]. The finding was similar to that of a previous feasibility study, which found that people with higher dementia knowledge and dementia attitude scores were more likely to continue to volunteer [43]. The levels of motivation could be affected by knowledge about dementia [16, 41] and reflected in the extent of an individual’s involvement in volunteer services [41]. Our findings support that the volunteer program could enhance dementia knowledge and attitudes, while these outcomes were found to be positively associated with the level of motivation to volunteer. This is an imperative implication to researchers and practitioners in the field to accelerate the related initiatives to cope with the dementia tsunami.

Taking on the role of being a volunteer in the community not solely better the person with dementia and the caregivers in the present moment, volunteering also offered them the opportunity to contemplate and be prepared in a state of readiness as they are aging. Jenkinson et al. [44] explored the development of volunteering as a public health promotion intervention. In comparison to those who do not engage or only episodically engage in voluntary work, continuously volunteering is associated with a significantly lower risk of dementia [45]. Recent research also supported that both self-oriented and other-oriented volunteering were significantly related to better health outcomes [46]. In a nationally representative sample of older adults, the initiation and maintenance of volunteering decreased the risk of cognitive impairment for over 14 years and provided the time to protect against the onset of cognitive impairment [47]. The knowledge and training may have sowed good seeds in the community and delicately nurtured in the process of volunteering. Thus, public health policymakers shall leverage the service of volunteers, and such efforts will potentially be returned in the long run from a public health perspective.

## Limitations

This study has a few limitations that deserve attention. First, as this study is a part of an implementation study, no control group had been designed to compare changes in dementia knowledge and attitudes. However, this is an imperative study that evaluated the effects of training and voluntary services on the volunteers’ dementia knowledge and attitudes, and the findings could provide important insights for the field of dementia care. Second, we were unable to delineate the effects of training/voluntary services because we measured knowledge and attitudes only at baseline and the end of the provision of services. Although the post-hoc multiple regression analysis results showed that the attendance of the training workshops and optional enrichment workshops, as well as the number of voluntary services delivered, were not associated with the magnitude of changes in knowledge and attitude among those who were qualified to provide services, it is suggested that knowledge and attitudes be assessed immediately after the training is provided, in order to track the changes more precisely. Third, we mainly relied on our partner organizations to recruit volunteers; thus, selection bias could not be eliminated. We suggest that in a future study, a more representative sample could be recruited. Fourth, this study cannot inform us which programme elements (i.e. training, enrichment workshop, voluntary services, peer support or others) contributed most to the attitude and knowledge change. Similarly, based on the findings, we could not suggest the optimal number of training sessions, enrichment workshops, and the number of services delivered that may change attitude and knowledge significantly. The abovementioned limitations were worth to be addressed in future research.

## Conclusions

Funding and providing health and social care for a growing aging population with an increasing number of chronic health conditions, such as dementia, are among the biggest challenges to global public health [48]. In the past three decades, the voluntary sector has been promoted as a cost-effective and locally responsive solution to providing home and community care for the aging population [49]. Numerous studies have been conducted to evaluate the impact of volunteer-delivered activities on people with dementia and their families, but this study is novel in that it highlights two important issues. A well-designed and continuous training program with plenty of practical experience is essential to improving the knowledge and attitudes towards dementia of volunteers. At the same time, knowledge and attitudes have been found to be related to the levels of motivation to volunteer. From our experience, despite educating and maintaining a team of active volunteers was not inexpensive, the changes on them and their contribution to the community can be long lasting. Volunteers are an essential resource in the community, and they might one day become a family caregiver or themselves a patient of dementia. More efforts have to be made to engage volunteers in dementia care.

## Data Availability

The datasets used and/or analyzed during the current study are available from the corresponding author on reasonable request.
